# Short- and Mid-Term Outcomes of Bovine Pericardial Patch vs. Saphenous Vein Patch in Femoral Endarterectomy

**DOI:** 10.3400/avd.oa.25-00146

**Published:** 2026-01-29

**Authors:** Takashi Harada, Daisuke Futagami, Yuki Echie, Saeki Watanabe, Hironobu Morimoto, Keijiro Katayama, Shogo Mukai, Masahito Taniguchi

**Affiliations:** 1 Department of Cardiovascular Surgery, Fukuyama Cardiovascular Hospital, Fukuyama, Hiroshima, Japan; 2 Department of Cardiology, Fukuyama Cardiovascular Hospital, Fukuyama, Hiroshima, Japan

**Keywords:** femoral endarterectomy, bovine pericardial patch, saphenous vein patch, endovascular treatment, peripheral artery disease

## Abstract

**Objectives:**

Femoral endarterectomy often requires patch angioplasty, and saphenous vein patch (SVP) was the standard. However, bovine pericardial patch (BPP) provides potential advantages, including no requirement for vein harvest, use of the access site for concomitant endovascular procedures, and favorable handling characteristics. We compared the short- and mid-term outcomes of BPP and SVP in femoral endarterectomy.

**Methods:**

This retrospective, single-center study included 42 patients (49 limbs) who underwent elective femoral endarterectomy with patch closure between September 2016 and January 2025. The patients were grouped by patch type as follows: 28 limbs with BPP and 21 limbs with SVP. Primary endpoints included patency and freedom from target lesion revascularization at the endarterectomy site. Secondary endpoints included perioperative complications, limb salvage, and intraoperative arterial clamp time.

**Results:**

No patch-site restenosis or re-intervention occurred in either group. There were no patch infections, and the perioperative complications were similar. Limb salvage at 40 months was 87.5% with BPP vs. 95.0% with SVP (p = 0.42). Intraoperative arterial clamp time was significantly shorter in the BPP group (55.0 vs. 69.5 min, p = 0.01).

**Conclusions:**

BPP represents a safe and valuable alternative option for femoral endarterectomy.

## Abbreviations

ABI
: ankle–brachial index
BPP
: bovine pericardial patch
CFA
: common femoral artery
CLI
: critical limb ischemia
CT
: computed tomography
DFA
: deep femoral artery
EVT
: endovascular treatment
FA
: femoral artery
PAD
: peripheral arterial disease
SFA
: superficial femoral artery
SVP
: saphenous vein patch
VA-ECMO
: veno-arterial extracorporeal membrane oxygenation

## Introduction

In recent years, revascularization of the iliac and superficial femoral arteries (SFAs) for peripheral arterial disease (PAD) has increased owing to advances in endovascular treatment (EVT) devices and techniques.^1,2)^ However, the common femoral artery (CFA) is considered a non-stenting zone due to the risk of stent fracture and its role as an access site for EVT; thus, balloon angioplasty alone yields suboptimal patency, and surgical endarterectomy is regarded as the first-line treatment.^3–7)^ Autologous veins or prosthetic grafts are commonly used as patch materials to prevent stenosis at the arteriotomy sites.^8)^

At our institution, a great saphenous vein patch (SVP) has been used owing to the risk of infection. Since 2021, we have been using a bovine pericardial patch (BPP) (XenoSure; LeMaitre Vascular, Burlington, MA, USA) for femoral endarterectomy. The advantages of BPP include preservation of the saphenous vein, reduced skin incisions, and ease of patch handling. In patients requiring concomitant EVT because of inadequate revascularization with endarterectomy alone, the patch also serves as a reliable access route. This study aimed to evaluate the short- and mid-term outcomes of BPP vs. SVP in femoral endarterectomy and to assess the utility and safety of BPP.

## Materials and Methods

This retrospective, single-center study at Fukuyama Cardiovascular Hospital was conducted to evaluate the clinical outcomes of femoral endarterectomy and was approved by the institutional review board (IRB #119). Written informed consent was obtained from all patients. This study included all the adult patients who underwent elective femoral endarterectomy. Patients who underwent emergency surgery for acute lower-limb arterial occlusion or femoral artery (FA) repair associated with veno-arterial extracorporeal membrane oxygenation (VA-ECMO) removal were excluded. The primary endpoints were patency and freedom from target lesion revascularization at the endarterectomy site, while the secondary endpoints included postoperative complications, freedom from major amputation, and intraoperative arterial clamp time.

**Fig. 1:**
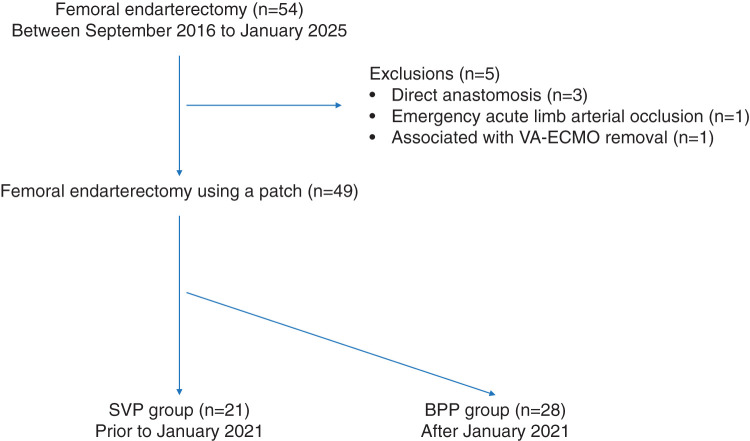
Flowchart of patient selection. VA-ECMO: veno-arterial extracorporeal membrane oxygenation; SVP: saphenous vein patch; BPP: bovine pericardial patch

Between September 2016 and January 2025, femoral endarterectomy using a patch was performed on 49 limbs in 42 patients with PAD of the FA. Of these, 23 were hybrid procedures involving concomitant EVT. Since 2021, after BPP became available, all surgical endarterectomies have been performed using BPP. Prior to 2021, when the lesion was confined to the FA, primary closure with continuous suturing was performed if the vessel diameter was ≥10 mm, whereas surgical endarterectomy using SVP was selected if the diameter was <10 mm. At our institution, primary closure is performed only in patients with an inherently sufficient vascular diameter in whom direct anastomosis does not result in narrowing compared with the typical FA size. We defined an adequate vascular diameter as ≥10 mm. Femoral endarterectomy was performed according to standard procedures in both the BPP and SVP groups. **Figure 1** shows the flowchart of patient selection. Preoperative computed tomography (CT) and ultrasonography were used to identify areas of wall thickening and calcification, followed by a skin incision after appropriate marking. In principle, the saphenous vein was harvested from the ipsilateral groin through the same incision. If additional length was required, a separate incision was made to harvest the SVP. The common, superficial, and deep femoral artery (DFA) were exposed to a sufficient length to ensure proper visualization of the lesion. After systemic heparinization, the artery was clamped and a longitudinal arteriotomy was performed. Clamping and arteriotomy sites were determined based on preoperative ultrasonography, CT, and intraoperative findings. Thromboendarterectomy was performed by completely removing the thickened intima, including organized thrombus and calcified lesions. The distal end of the intima was then tackled using several stitches using 6-0 polypropylene sutures. Subsequently, either the BPP or SVP was trimmed and anastomosed using continuous 6-0 polypropylene sutures. The smooth surface of the BPP was oriented toward the vascular lumen.

**Fig. 2:**
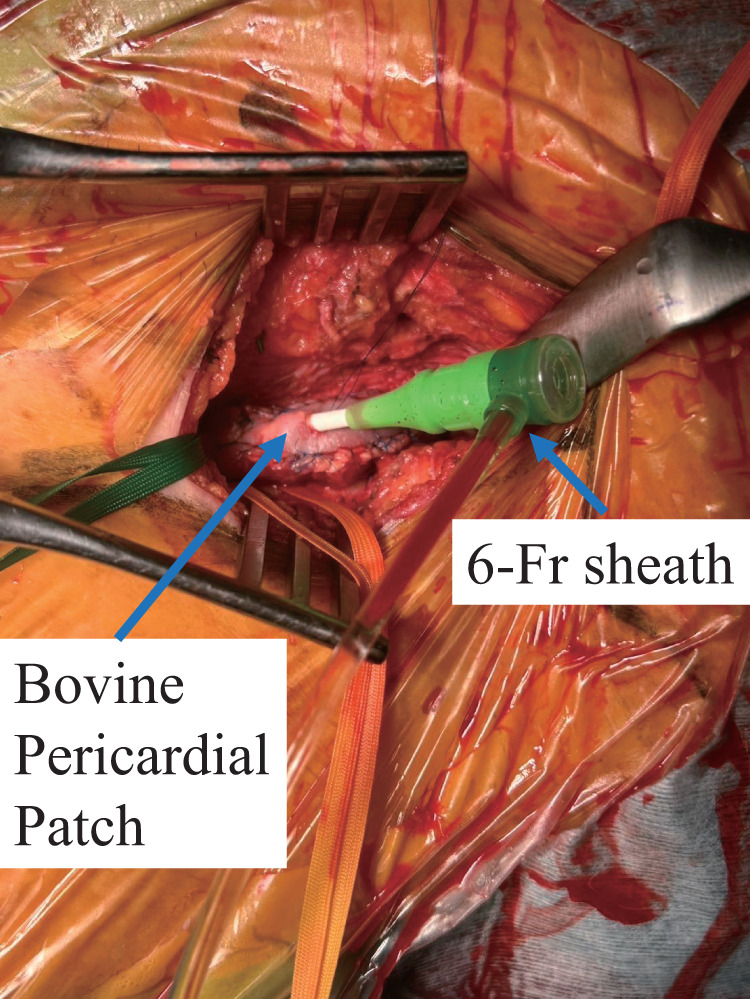
Femoral endarterectomy using a BPP. A BPP was sutured to the common femoral artery and superficial femoral artery with a 6-0 polypropylene suture. BPP also serves as a useful access route for EVT, allowing the insertion of a 6-Fr sheath. The sheath fits tightly, resulting in no bleeding or displacement during the procedure. BPP: bovine pericardial patch; EVT: endovascular treatment

Hybrid procedures with concomitant EVT were performed when significant concomitant lesions were present in the iliac artery or the ipsilateral SFA-to-popliteal artery requiring revascularization. For iliac artery lesions, vascular access was obtained via the upper extremity. For ipsilateral SFA-to-popliteal artery lesions, in the BPP group, a U-stitch using 6-0 polypropylene was placed on the patch, and a 6-Fr sheath was inserted through the patch to perform ipsilateral EVT (**Fig. 2**), whereas in the SVP group, the patch was not punctured and a sheath was inserted directly through the arteriotomy under arterial clamping. All hybrid procedures were planned preoperatively and performed concomitantly with femoral endarterectomy.

All patients underwent in-hospital evaluations, including ankle–brachial index (ABI) measurements and CT or ultrasonography. In the outpatient follow-up, ABI and Duplex ultrasound examinations were performed every 6 months. Duplex ultrasonography was used as the primary assessment modality, and CT was performed in patients in whom ultrasonographic evaluation was not feasible and who exhibited clinical symptoms or a decrease in the ABI. Restenosis was defined as a peak systolic velocity ratio ≥2.8 on Duplex ultrasonography or ≥50% luminal narrowing on follow-up CT or computed angiography. Additional reinterventions were performed at the discretion of the treating physician. Patients initially received dual antiplatelet therapy postoperatively, which was reduced to a single antiplatelet after 6 months. Those already on anticoagulation were treated postoperatively with both an anticoagulant and an antiplatelet agent, followed by anticoagulant monotherapy after 6 months.

The mean follow-up period was 39 ± 28.9 months overall, 18 ± 15.4 months in the BPP group, and 67 ± 16.2 months in the SVP group (p <0.01). Patients who were lost to outpatient follow-up were contacted via telephone to obtain the necessary data. The follow-up rate was 100%.

### Statistical analysis

Statistical analyses were performed using EZR software (Saitama Medical Center, Jichi Medical University, Saitama, Japan). Continuous data are presented as mean ± standard deviation. Continuous variables following a normal distribution were analyzed using the t-test, whereas those that did not follow a normal distribution were assessed using the Mann–Whitney U test. Categorical variables were analyzed using Fisher’s exact test.

Event-free curves were estimated using the Kaplan–Meier method and compared using the log-rank test. In addition to the primary analysis, an exploratory subgroup analysis was performed to compare perioperative and mid-term outcomes between pure open and hybrid surgery. The detailed results of the subgroup analysis are provided in the Supplementary Materials. p <0.05 was considered to be statistically significant.

## Results

During the study period, 47 patients (54 limbs) underwent femoral endarterectomy and angioplasty. Three patients (3 limbs) who underwent direct closure, 1 patient (1 limb) with emergency acute limb arterial occlusion, and 1 patient (1 limb) associated with VA-ECMO removal were excluded from the study. A total of 42 patients (49 limbs) were ultimately included in the study and divided into 2 groups: 28 limbs in the BPP group and 21 limbs in the SVP group (**Fig. 1**).

The mean age at the time of surgery was 72.9 ± 11.6 years, and the majority of patients (81.6%) were male. **Table 1** shows the preoperative characteristics and cardiovascular risk factors of each group, reflecting the typical features of patients with atherosclerotic diseases.

**Table 1:** Preoperative characteristics and intraoperative data

	Overall (n = 49)	BPP (n = 28)	SVP (n = 21)	p-Value
Characteristics				
Age (years)	72.9 ± 11.6	74.1 ± 10.0	71.3 ± 13.4	0.42
Male (n)	40 (81.6%)	21 (75.0%)	19 (90.5%)	0.27
Comorbidity				
Hypertension (n)	43 (87.8%)	25 (89.3%)	18 (85.7%)	1
Diabetes (n)	34 (69.4%)	20 (71.4%)	14 (66.7%)	0.76
Dyslipidemia (n)	26 (53.1%)	14 (50%)	12 (57.1%)	0.77
CAD (n)	22 (44.9%)	13 (46.4%)	9 (42.9%)	1
CKD (eGFR<40) (n)	13 (26.5%)	9 (32.1%)	4 (19.0%)	0.35
ESRF on HD (n)	10 (20.4%)	8 (28.6)	2 (9.5%)	0.16
COPD (n)	6 (12.2%)	3 (10.7%)	3 (14.3%)	1
Smoking history (n)	35 (71.4%)	22 (78.6%)	13 (61.9%)	0.22
Current smoker (n)	17 (34.7%)	8 (28.6%)	9 (42.9%)	0.37
Cerebrovascular disease (n)	17 (35.0%)	12 (42.9%)	5 (23.8%)	0.23
History of intervention for ASO (n)	24 (49.0%)	15 (53.6%)	9 (42.9%)	0.57
CLI (n)	10 (20.4%)	6 (21.4%)	4 (19.0%)	1
Medication				
Antiplatelet agents (n)	44 (89.8%)	26 (92.9%)	18 (85.7%)	0.64
Anticoagulants (n)	11 (22.4%)	5 (17.9%)	6 (28.6%)	0.49
Intraoperative data				
Operative time (min)	174.5 ± 60.9	174 ± 61.7	175.2 ± 61.5	0.95
Operative time excluded EVT (min)	142.1 ± 36.5	136.7 ± 37.4	148.9 ± 35.0	0.26
Clamp time (min)	61.1 ± 19.4	55.0 ± 14.0	69.5 ± 22.7	<0.01
Lesion location				
CFA alone (n)	32 (65.3%)	18 (64.3%)	14 (66.7%)	1
CFA and SFA (n)	15 (30.6%)	9 (32.1%)	6 (28.6%)	1
SFA alone (n)	2 (4.1%)	1 (3.6%)	1 (4.8%)	1
DFA	13 (26.5%)	8 (28.6%)	5 (23.8%)	0.76
Combined procedure				
EVT (n)	23 (46.9%)	15 (53.6%)	8 (38.1%)	0.39
Ipsilateral				
Iliac EVT (n)	10 (20.4%)	5 (17.9%)	5 (23.8%)	0.73
SFA EVT (n)	12 (24.5)	8 (28.6%)	4 (19.0%)	0.52
Popliteal EVT (n)	3 (6.1)	3 (10.7%)	0 (0.0%)	0.25
Contralateral				
Iliac EVT (n)	5 (10.2)	3 (10.7%)	2 (9.5%)	1
Patch length (cm)	5.1 ± 1.7	5.5 ± 1.9	4.5 ± 1.0	0.063
Blood loss (mL)	182.4 ± 174.6	201.2 ± 191.4	159 ± 152.6	0.43

BPP: bovine pericardial patch; SVP: saphenous vein patch; CAD: coronary artery disease; CKD: chronic kidney disease; eGFR: estimated glomerular filtration rate; ESRF: end-stage renal failure; HD: hemodialysis; COPD: chronic obstructive pulmonary disease; ASO: arteriosclerosis obliterans; CLI: critical limb ischemia; EVT: endovascular treatment; CFA: common femoral artery; SFA: superficial femoral artery; DFA: deep femoral artery

Overall, 20.4% of patients had critical limb ischemia (CLI), with no statistically significant differences between the 2 groups.

**Table 1** also shows the intraoperative data for each group. All femoral endarterectomy procedures were performed under general anesthesia. Operative time and operative time excluding EVT were comparable between the BPP and SVP groups (174.6 ± 61.7 vs. 175.2 ± 61.5 min, p = 0.95; 136.7 ± 37.4 vs. 148.9 ± 35.0 min, p = 0.26). The arterial clamp time was significantly shorter in the BPP group than in the SVP group (55.0 ± 14.0 vs. 69.5 ± 22.7 min, p <0.01). The lesion location, concomitant EVT rate, and distribution of target vessels did not differ significantly between the groups. Patch length tended to be longer in the BPP group, although this did not reach statistical significance (5.5 ± 1.9 vs. 4.5 ± 1.0 cm, p = 0.063). Estimated blood loss was also comparable between the groups.

**Table 2** summarizes the postoperative complications and mid-term outcomes. No perioperative death, bleeding events, patch infections, or remote infections occurred in either group. Wound complications occurred in 12.2% of patients overall (14.3% in the BPP group vs. 9.5% in the SVP group, p = 0.69). All wound complications resolved with antibiotic therapy or superficial debridement, and none required patch removal. Peripheral neuropathy, including numbness or paresthesia, was observed in 5 patients overall (10.2%): 2 (7.1%) in the BPP group and 3 (14.3%) in the SVP group (p = 0.64). All cases were mild and transient. Coronary artery disease-related events were rare (2.0% overall). The mean follow-up period was significantly longer in the SVP group than in the BPP group (67.0 ± 16.2 vs. 18.0 ± 15.4 months, p <0.01). During the follow-up period, additional EVT was required in 16 patients for lesions outside the femoral endarterectomy site, including 9 patients in the BPP group and 7 in the SVP group.

**Table 2:** Postoperative complications and mid-term outcome

	Overall (n = 49)	BPP (n = 28)	SVP (n = 21)	p-Value
Postoperative complication				
Perioperative death	0	0	0	
Wound complication	6 (12.2%)	4 (14.3%)	2 (9.5%)	0.69
Bleeding	0	0	0	
CAD	1 (2.0%)	0	1 (4.8%)	0.43
Neurological	5 (10.2%)	2 (7.1%)	3 (14.3%)	0.64
Patch infection	0	0	0	
Remote infection	0	0	0	
Mid-term outcome				
Follow-up period (month)	39.0 ± 28.9	18.0 ± 15.4	67.0 ± 16.2	<0.01
ABI				
Pre-operation	0.46 ± 0.28	0.45 ± 0.29	0.48 ± 0.26	0.69
After surgery (1 week)	0.92 ± 0.19	0.95 ± 0.15	0.89 ± 0.23	0.31
Major limb amputation	2	1	1	0.42
Restenosis of CFE	0	0	0	
Patch enlargement	1	1	0	0.3
Additional intervention of EVT	16	9	7	0.089

BPP: bovine pericardial patch; SVP: saphenous vein patch; CAD: coronary artery disease; ABI: ankle–brachial index; CFE: common femoral artery; EVT: endovascular treatment

**Fig. 3:**
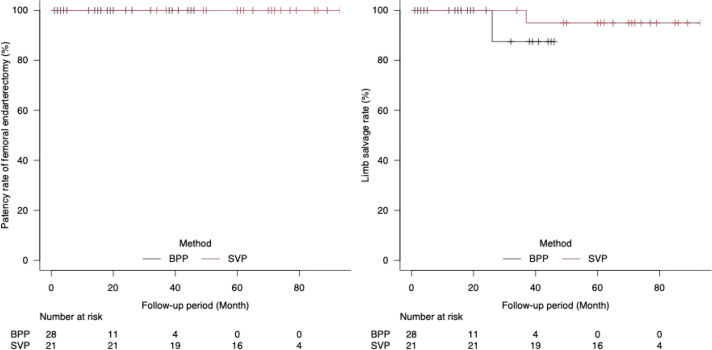
(**A**) Primary patency rate after femoral endarterectomy. There were no cases of restenosis at the patch site, and no reinterventions were required during the follow-up period. (**B**) Limb salvage rate after femoral endarterectomy. Limb salvage was 100% in both groups at 20 months. At 40 months, it was 87.5% (95% CI: 0.39–0.98) in the BPP group and 95.0% (95% CI: 0.70–0.99) in the SVP group, with no statistically significant difference between the groups (p = 0.42). CI: confidence interval; BPP: bovine pericardial patch; SVP: saphenous vein patch

There were no cases of restenosis at the patch site, and no reinterventions were required during the follow-up period (**Fig. 3A**).

**Figure 3B** shows the limb salvage rates after femoral endarterectomy in the BPP and SVP groups. Limb salvage rate was 100% in both groups at 20 months. At 40 months, it was 87.5% (95% confidence interval [CI]: 0.39–0.98) and 95.0% (95% CI: 0.70–0.99) in the BPP and SVP groups, respectively, with no statistically significant difference between the groups (p = 0.42). Despite the patency of the femoral endarterectomy site, 1 limb in the BPP group was unsalvageable 27 months after femoral endarterectomy due to incomplete foot ischemia with popliteal obstruction and poor general condition. In the SVP group, 1 limb required major limb amputation 37 months after femoral endarterectomy due to the progression of arteriosclerosis from the SFA to the below-knee arteries with deterioration in the general condition. CLI was observed in both patients.

In the subgroup analysis comparing pure open and hybrid surgery, preoperative patient characteristics in the hybrid surgery group had a significantly higher prevalence of prior interventions for arteriosclerosis obliterans (ASO) than those in the pure open group (pure open vs. hybrid surgery: 9/26, 34.6% vs. 15/23, 65.2%; p = 0.047), as well as a significantly lower preoperative ABI (0.56 ± 0.23 vs. 0.35 ± 0.29; p <0.01). Nevertheless, favorable outcomes were maintained in both subgroups, with no significant differences in perioperative complications or mid-term outcomes (**Supplementary Tables 1** and **2**).

## Discussion

EVT has become the first-line option for iliac and SFA lesions due to technological advancements and its minimally invasive nature.^1,2)^ Endarterectomy remains the standard treatment for CFA lesions.^3–7)^

This single-center retrospective study evaluated and compared the short- and mid-term outcomes of BPP and SVP for femoral endarterectomy. During follow-up, no restenosis or re-intervention at the patch site occurred in either group. There were no statistically significant differences between patch types pertaining to perioperative complications, including infections and major limb amputation.

The short- and mid-term efficacy of BPP with femoral endarterectomy has been reported, and it is considered a promising material for vascular reconstruction.^9–13)^ In the carotid artery region, BPP has demonstrated favorable antithrombotic properties and durability, with long-term outcomes comparable to those of SVP and synthetic graft patch.^14–16)^

When an autologous vein is used, additional or extended skin incisions are needed for graft harvesting. Patients with PAD often have concomitant coronary artery disease and may require future bypass surgery; thus, preserving the saphenous vein as a potential graft is often considered.^17)^ In the present study, 16 limbs (33%) underwent additional EVT for lesions outside the patch repair site during follow-up after femoral endarterectomy. These findings suggest that using BPP may help preserve autologous graft options for future bypass procedures. In the BPP group, concomitant EVT was performed in 15 limbs (53.6%), underscoring the patch’s suitability as an access site for contemporary hybrid revascularization. Its excellent hemostatic properties, ease of handling, and adequate length enable rapid and secure closure, potentially shortening arterial clamp time during combined procedures. In this study, the shorter intraoperative arterial clamp time observed in the BPP group may be explained by differences in the procedural approach during concomitant EVT. When EVT was performed intraoperatively on the ipsilateral distal side to the SFA, the BPP group underwent EVT via puncture of the patch after clamp release, whereas in the SVP group, EVT was performed through sheath insertion from the arteriotomy site under continued arterial clamping. This approach also shortens the arterial clamp time and is considered beneficial for ischemic limbs, which generally have limited tolerance to ischemia. Given the increasing number of high-risk patients with complex ASO lesions in an aging society, the demand for hybrid procedures continues to rise. Subgroup analyses (**Supplementary Tables 1** and **2**) demonstrated that the hybrid surgery group was characterized by a higher prevalence of prior interventions for ASO as well as a significantly lower preoperative ABI, indicating a more advanced disease severity. Nevertheless, favorable outcomes were maintained in both subgroups, with no significant differences in postoperative complications and mid-term outcomes. These findings further support the feasibility and safety of the hybrid strategy, even in patients with more severe ischemic disease. In the present study, hybrid procedures accounted for 47.0% of all cases, highlighting the clinical relevance of this strategy in contemporary practice. Providing a stable access route with the BPP represents a significant advantage when concomitant EVT is required.

Polyesters and other synthetic patch materials are associated with a risk of prosthetic infection. BPP is a biological material, and its resistance to infection has been suggested in several reports regarding its use in infectious arterial aneurysms and dialysis graft infections in the cardiovascular field.^18,19)^ In this study, no patch infections were observed in the BPP group. The wound infections were successfully managed with antibiotics and superficial debridement alone, and none of the patients required patch removal. From a practical perspective, although BPP is associated with higher material costs, it provides consistent material availability with standardized size and quality.

Both the BPP and SVP groups showed favorable outcomes with no patch-site restenosis or patch infection. Satisfactory limb salvage rates were achieved in both groups. Considering its advantages, such as preservation of the saphenous vein, accessibility for hybrid procedures, ease of handling, reduction of arterial clamp time, and infection resistance, BPP is expected to be a valuable alternative option for femoral endarterectomy.

### Limitations

This study was a single-center, retrospective analysis and provided a lower level of evidence than prospective investigations. Our sample size was small, and observation was limited to the short- and mid-term; larger studies with long-term follow-up are required to validate these findings.

As the BPP was adopted only after 2021, the median follow-up duration differs significantly between groups. To account for the differing observation periods, mid-term outcomes were analyzed using Kaplan–Meier estimates with log-rank tests. Cox regression was not conducted because of the limited number of events, which could have led to model overfitting.

Differences in the observation period may have been associated with surgeon learning curves and increasing experience with endarterectomy, which may have influenced the outcomes and should be considered a potential source of bias. However, over the entire study period, perioperative management, diagnostic criteria, and surgical basic anastomotic techniques were applied consistently. The procedural difference was that, in the BPP group, EVT for lesions distal to the SFA was performed by puncturing the patch after completion of the patch anastomosis and release of the arterial clamp.

## Conclusions

This retrospective study found no significant differences between BPP and SVP groups in terms of short- and mid-term outcomes, including patch restenosis, major lower limb amputation, and perioperative complications, including patch infection. Additionally, BPP application preserves the saphenous vein and facilitates vascular access for concomitant EVT during hybrid procedures, making it a useful option for femoral endarterectomy. Therefore, further long-term studies involving larger patient cohorts are warranted.

## Supplementary Materials

Supplementary Table 1 Preoperative characteristics and intraoperative data in pure open and hybrid surgery.

Supplementary Table 2 Postoperative complication and mid-term outcome in pure open and hybrid surgery.

## References

[1] Ichihashi S, Higashiura W, Itoh H, et al. Long-term outcomes for systematic primary stent placement in complex iliac artery occlusive disease classified according to Trans-Atlantic Inter-Society Consensus (TASC)-II. J Vasc Surg 2011; 53: 992–9. 21215582 10.1016/j.jvs.2010.10.069

[2] Katsuki T, Yamaji K, Hiramori S, et al. Ten-year clinical outcomes for patients undergoing lower extremity endovascular interventions. J Vasc Surg 2020; 72: 1626–35.e3. 32278575 10.1016/j.jvs.2020.02.026

[3] Nakama T, Takahara M, Iwata Y, et al. 1-year outcomes of thromboendarterectomy vs endovascular therapy for common femoral artery lesions: CAULIFLOWER study results. JACC Cardiovasc Interv 2022; 15: 1453–63. 35863795 10.1016/j.jcin.2022.03.010

[4] Kuma S, Tanaka K, Ohmine T, et al. Clinical outcome of surgical endarterectomy for common femoral artery occlusive disease. Circ J 2016; 80: 964–9. 26902450 10.1253/circj.CJ-15-1177

[5] Hashimoto T, Yamamoto S, Kimura M, et al. Long-term outcomes following common femoral endarterectomy. J Clin Med 2022; 11: 6873. 36431350 10.3390/jcm11226873 PMC9697575

[6] Troisi N, Bertagna G, Artini V, et al. Open surgery of common femoral artery occlusive disease: a contemporary review. J Cardiovasc Surg (Torino) 2024; 65: 324–9. 10.23736/S0021-9509.24.13098-4 38896089

[7] Elbadawy A, Ali H, Saleh M. Midterm outcomes of common femoral endarterectomy combined with inflow and outflow endovascular treatment for chronic limb threatening ischaemia. Eur J Vasc Endovasc Surg 2020; 59: 947–55. 32224037 10.1016/j.ejvs.2020.02.028

[8] Kobayashi T, Takahara M, Fujimura N, et al. Comparison of clinical outcomes in patients undergoing common femoral thromboendarterectomy with or without patch angioplasty. Eur J Vasc Endovasc Surg 2023; 65: 870–7. 36967011 10.1016/j.ejvs.2023.03.034

[9] Okazaki T, Kobayashi T, Mochizuki S, et al. Clinical outcomes of common femoral thromboendarterectomy with bovine pericardium patch angioplasty. Ann Vasc Surg 2024; 98: 194–200. 37385339 10.1016/j.avsg.2023.06.010

[10] Okadome J, Morisaki K, Matsuda D, et al. Comparison of early outcomes in patients who underwent common femoral thromboendarterectomy with vein versus bovine pericardial patches. Ann Vasc Surg 2025; 110: 498–504. 39424177 10.1016/j.avsg.2024.08.032

[11] Ren L, Sun Y, Wang H, et al. Mid-term outcomes of common femoral patch angioplasty in iliofemoral occlusive diseases: bovine pericardial patch versus great saphenous vein patch. Ann Vasc Surg 2025; 114: 106–15. 39880282 10.1016/j.avsg.2024.12.075

[12] Li X, Guo Y, Ziegler KR, et al. Current usage and future directions for the bovine pericardial patch. Ann Vasc Surg 2011; 25: 561–8. 21276709 10.1016/j.avsg.2010.11.007 PMC3085588

[13] Noronen K, Söderström M, Kouhia S, et al. Bovine pericardial patch: a good alternative in femoral angioplasty. J Vasc Surg 2023; 77: 225–30. 35987464 10.1016/j.jvs.2022.08.010

[14] Ho KJ, Nguyen LL, Menard MT. Intermediate-term outcome of carotid endarterectomy with bovine pericardial patch closure compared with Dacron patch and primary closure. J Vasc Surg 2012; 55: 708–14. 22226180 10.1016/j.jvs.2011.10.007

[15] Liesker DJ, Gareb B, Looman RS, et al. Patch angioplasty during carotid endarterectomy using different materials has similar clinical outcomes. J Vasc Surg 2023; 77: 559–66.e1. 36208708 10.1016/j.jvs.2022.09.027

[16] Léonore FT, Elsa FT, David PC, et al. Short- and long-term outcomes following biological pericardium patches versus prosthetic patches for carotid endarterectomy: a retrospective bicentric study. Ann Vasc Surg 2021; 72: 66–71. 32339685 10.1016/j.avsg.2020.04.010

[17] Yamamoto Y, Uchiyama H, Oonuki M. Outcomes of femoral endarterectomy with superficial tributary vein patch angioplasty. Ann Vasc Surg 2023; 90: 197–203. 36473670 10.1016/j.avsg.2022.10.026

[18] McMillan WD, Leville CD, Hile CN. Bovine pericardial patch repair in infected fields. J Vasc Surg 2012; 55: 1712–5. 22459752 10.1016/j.jvs.2011.11.139

[19] Anibueze C, Sankaran V, Sadat U, et al. Neoaortic xenoprosthetic grafts for treatment of mycotic aneurysms and infected aortic grafts. Ann Vasc Surg 2017; 44: 419.e1–e12. 10.1016/j.avsg.2017.02.021 28642109

