# Supplementary material for: Short- and Mid-Term Outcomes of Bovine Pericardial Patch vs. Saphenous Vein Patch in Femoral Endarterectomy
Source: Ann Vasc Dis. 2026 Jan 29;19(1):25-00146. doi: 10.3400/avd.oa.25-00146 (PMC12864046; doi:10.3400/avd.oa.25-00146)
Supplement: Supplementary Table 1 — Preoperative characteristics and intraoperative data in pure open and hybrid surgery. [file avd-19-1-25-00146-s001.pdf]

Supplmentary Table 1 Preoperative characteristics and intraoperative data in pure open and hybrid surgery

Characteristics	Pure open(n=26)	Hybrid surgery(n=23)	P-Value
Age (years)	74.3±12.3	71.3±10.7	0.38
Male (n)	20(76.9%)	20(87.0%)	0.47
Comorbidity			
Hypertension (n)	23(88.4%)	20(87.0%)	1
Diabetes (n)	19(73.1%)	15(65.2%)	0.76
Dyslipidemia (n)	16(61.5%)	10(43.5%)	0.26
CAD (n)	12(46.2%)	10(43.5%)	0.47
CKD(eGFR＜40) (n)	6(23.1%)	7(30.4%)	0.75
ESRF on HD (n)	5(19.2%)	5(21.7%)	1
COPD (n)	2(7.7%)	4(17.4%)	0.4
Smoking history (n)	14(53.8%)	21(91.3%)	<0.01
Current smoker (n)	6(23.1%)	11(47.8%)	0.082
Cerebrovascular disease (n)	6(23.1%)	11(47.8%)	0.082
History of intervention for ASO (n)	9(34.6%)	15(65.2%)	0.047
CLI (n)	4(15.4%)	6(26.1%)	0.48
Medication			
Antiplatelet agents (n)	21(80.8%)	23(100%)	0.052
Anticoagulants (n)	6(23.1%)	5(21.7%)	1
Intraoeprative data			
SVP use	13(50.0%)	8(34.8%)	0.39
BPP use	13(50.0%)	15(65.2%)	0.39
Operative time (min)	138.0±29.8	215.8±61.2	<0.01
EVT time (min)		69.9±44.0	
Clamp time (min)	56.2±14.6	66.7±22.7	0.064
Lesion location			
CFA alone (n)	20(76.9%)	12(52.2%)	0.082
CFA and SFA (n)	4(15.4%)	11(47.8%)	0.028
SFA alone (n)	2(7.7%)	0	0.49
DFA	5(19.2%)	8(34.8%)	0.33
Patch length (cm)	4.8±1.1	5.4±2.2	0.27
Blood loss (ml)	131.6±86.2	159±152.6	0.027

Supplementary Table 2 Postoperative complication and mid-term outcome in pure open and hybrid surgery

Postoperative complication	Pure open(n=26)	Hybrid surgery(n=23)	P-Value
Perioperative death	0	0	
Wound complication	1(3.8%)	5 (21.7%)	0.086
Bleeding	0	0	
CAD	0	1 (4.4%)	0.47
Neurological	3(11.5%)	2(8.7%)	1
Patch infection	0	0	
Remote infection	0	0	
Mid-term outcome			
Follow-up period（month）	37.4.±37.3	34.4±20.8	0.74
ABI			
Pre operation	0.56±0.23	0.35±0.29	<0.01
After surgery (1week)	0.97±0.15	0.87±0.23	0.071
Major limb amputation	1(3.8%)	1(4.3%)	1
Restenosis of CFE	0	0	
Patch enlargement	1(3.8%)	0	1
Additional intervention of EVT	6(23.1%)	10(43.5%)	0.23
